# Remote psychophysical evaluation of olfactory and gustatory functions in early-stage coronavirus disease 2019 patients: the Bologna experience of 300 cases

**DOI:** 10.1017/S0022215120001358

**Published:** 2020-07-01

**Authors:** M Petrocelli, F Ruggiero, A M Baietti, P Pandolfi, G Salzano, F A Salzano, J R Lechien, S Saussez, G De Riu, L A Vaira

**Affiliations:** 1 Maxillofacial Surgery Operative Unit, Bellaria-Maggiore Hospital, Azienda Unità Sanitaria Locale di Bologna, Italy; 2 Maxillofacial Surgery School, University Hospital of Parma, Bologna, Italy; 3 Department of Hygiene, Epidemiology and Public Health, Azienda Unità Sanitaria Locale di Bologna, Italy; 4 Maxillofacial Surgery Unit, University Hospital of Naples ‘Federico II’, Italy; 5 Otolaryngology Operative Unit, Department of Medicine, Surgery and Dentistry, ‘Scuola Medica Salernitana’, University of Salerno, Italy; 6 COVID-19 Task Force of the Young-Otolaryngologists of the International Federation of Oto-rhino-laryngological Societies (‘YO-IFOS’), Belgium; 7 Department of Human and Experimental Oncology, Faculty of Medicine, University of Mons (‘UMONS’) Research Institute for Health Sciences and Technology, Belgium; 8 Maxillofacial Surgery Operative Unit, University Hospital of Sassari, Italy

**Keywords:** COVID-19, Taste, Smell, Anosmia, Ageusia, SARS-CoV-2 Infection, Coronavirus

## Abstract

**Background:**

An objective evaluation of coronavirus disease 2019 in the first days of infection is almost impossible, as affected individuals are generally in home quarantine, and there is limited accessibility for the operator who should perform the test. To overcome this limitation, a recently validated psychophysical self-administered test was used, which can be performed remotely in the assessment of early-stage coronavirus disease 2019 patients.

**Methods:**

Olfactory and gustatory functions were objectively assessed in 300 patients in the first 7 days from coronavirus disease 2019 symptom onset.

**Results:**

Seventy per cent of the patients presented olfactory and/or gustatory disorders. The dysfunctions detected were mainly complete anosmia (47 per cent) or ageusia (38 per cent). A significant correlation was found between taste dysfunction and female gender (odds ratio = 1.936, *p* = 0.014) and fever (odds ratio = 2.132, *p* = 0.003).

**Conclusion:**

The psychophysical evaluation protocol proposed is an effective tool for the fast and objective evaluation of patients in the early stages of coronavirus disease 2019. Chemosensitive disorders have been confirmed to be frequent and early symptoms of the coronavirus infection, and, in a significant number of cases, they are the first or only manifestation of coronavirus disease 2019.

## Introduction

The high frequency of chemosensitive disorders in patients with coronavirus disease 2019 (Covid-19) is a clinical finding reported by several authors in Europe and America.^1–10^ These symptoms are typical of the early stages of the disease,^11,12^ and, considering their specificity, they may be useful as a screening marker.^13,14^

Psychophysical tests are crucial to determine the exact frequency, extent and clinical characteristics of these chemosensitive disorders, and to monitor their recovery over time. However, most of the studies currently present in the literature are based on anamnestic or observational evidence. As of 25 May 2020, only four objective studies had been published.^15–18^ These few studies mainly include patients with advanced stages of Covid-19 at the time of worst impairment, preventing the evaluation of chemosensitive functions. An objective evaluation in the first days of the disease is almost impossible, as these patients are generally in home quarantine, and there is limited accessibility for the operator who should perform the test.

A few weeks ago, Vaira *et al*.^19^ proposed and validated a new self-administered psychophysical test that can be performed remotely for the evaluation of olfactory and gustatory functions in patients in home quarantine. Using this test, we have objectively evaluated the chemosensitive functions of 300 Covid-19 patients, belonging to the healthcare staff of the Bellaria-Maggiore Hospital in Bologna, within the first 7 days of Covid-19 clinical onset.

## Materials and methods

This cohort study was conducted on severe acute respiratory syndrome 2 (SARS-CoV-2) positive patients monitored by the Surveillance and Prevention Department of Bellaria-Maggiore Hospital in Bologna, between 16 April and 2 May 2020. All the subjects were healthcare staff at the hospital.

In order to be eligible for enrolment in this study, patients had to meet the following inclusion criteria: adult over 18 years of age; SARS-CoV-2 infection confirmed by nasopharyngeal swab; and Covid-19 clinical onset (or positive swab in asymptomatic patients) less than 7 days previously.

Patients were excluded from the study if they did not answer the phone call twice, refused to participate, or presented a history of: previous gustatory or olfactory disorders, chronic rhinosinusitis, surgery, or radiotherapy or trauma to the oral and nasal cavities. The study protocol was approved by the Azienda Unità Sanitaria Locale di Bologna Ethical Committee (number: 378-2020-OSS-AUSLBO).

The lists of SARS-CoV-2 positive subjects were obtained from the Surveillance and Prevention Department of Bellaria-Maggiore Hospital. Two operators called each individual from the lists within 7 days of Covid-19 clinical onset.

Each telephone interview started with the request for the patient's explicit consent to participate in the study. Next, the patient's demographics (i.e. age, gender and professional role), co-morbidities or conditions that could be a cause for exclusion from the study were recorded. The presence of other Covid-19 symptoms and the date of onset were also recorded for each patient.

Finally, the chemosensitive psychophysical test procedure was explained to the subjects. Specifically, the patients were asked to collect seven common household odorants and prepare four basic flavoured solutions, before calling the operator again to perform the test. The test methodology and the scoring system have already been described by Vaira *et al*.^19^ Both the olfactory and gustatory functions were objectively evaluated.

The olfactory threshold was established by means of solutions with increasing dilutions of denatured ethyl-alcohol. Each patient was asked to prepare a 40 per cent solution of ethyl-alcohol in 100 ml water (bottle 0) using a syringe or a graduated container. In the subsequent bottles (bottles 1–8), the patients diluted a part of the previous solution with two parts of water, thus obtaining solutions with consequent 1:3 dilutions. A bottle filled with water was used as control. The test was performed by asking the patients to smell the alcohol-containing bottles, starting with the least concentrated (bottle 8), reporting if they found any differences compared to the control bottle. In the case of a negative answer, the examination continued with the subsequent bottle.^19^

The discriminative test was performed by asking each patient to collect some common household odorants: group A – orange juice, lemon juice or other citrus fruit juice; group B – pepper, rosemary, bay leaf or sage; group C – ‘Marseille’ or neutral soap; group D – wine or vinegar; group E – chocolate, Nutella® or coffee; and group F – Vicks VapoRub®, mint toothpaste or mouthwash. The subjects were then asked to smell one odorant from each group, giving a score reflecting their discriminative ability from 0 (no discrimination) to 10 (normal discrimination). The discriminative score was obtained from the average of the ratings for the six odorants.

The olfactory threshold and identification test scores were summed to provide an overall composite score; this allows a clinical classification of olfactory function (Table 1).^19^

**Table 1.** Self-administered chemosensory test scoring systems: olfactory test

Olfactory threshold score	Olfactory threshold composite score	Odour discrimination score	Odour discrimination composite score	Overall composite score*	Clinical diagnosis
7–8	50	8–10	50	90–100	Normal
6	40	6–7.9	40	70–80	Mild hyposmia
5	30	4–5.9	30	50–60	Moderate hyposmia
4	20	3–3.9	20	20–40	Severe hyposmia
2–3	10	1–2.9	10	0–10	Anosmia
0–1	0	0–0.9	0	90–100	Normal

* Olfactory threshold plus odour discrimination scores

The gustatory function was assessed by means of four solutions, one for each primary taste. These were prepared by each patient as follows: sweet solution – 60 g of refined sugar in 1 litre of water; sour solution – 90 ml of 100 per cent lemon juice in 1 litre of water; salted solution – 30 g of table salt in 1 litre of water; and bitter solution – unsweetened decaffeinated coffee.^19^ The patients were asked to taste a teaspoon of each solution, scoring the quality of their taste perception from 0 (ageusia) to 10 (normal perception). The bitter solution was always administered last. The gustatory score was obtained by averaging the values reported for each of the primary tastes (Table 2).

**Table 2.** Self-administered chemosensory test scoring systems: gustatory test

Patient administered scoring	Taste scoring system	Clinical diagnosis
10–7	4	Normal
5–6.9	3	Mild hypogeusia
3–4.9	2	Moderate hypogeusia
1–2.9	1	Severe hypogeusia
0–0.9	0	Ageusia

Statistical analysis was performed using SPSS software, version 26.0 (IBM, Armonk, New York, USA). Categorical variables are reported in terms of numerals and percentages of the total. Descriptive statistics for quantitative variables are given as the mean ± standard deviation. Logistic regression analysis and the Fisher's exact test were used to evaluate the significance of the correlations between the chemosensitive disorders and the general and clinical variables. The level of statistical significance was set at *p* ≤ 0.05, with a 95 per cent confidence interval (CI).

## Results

**Fig. 1.**
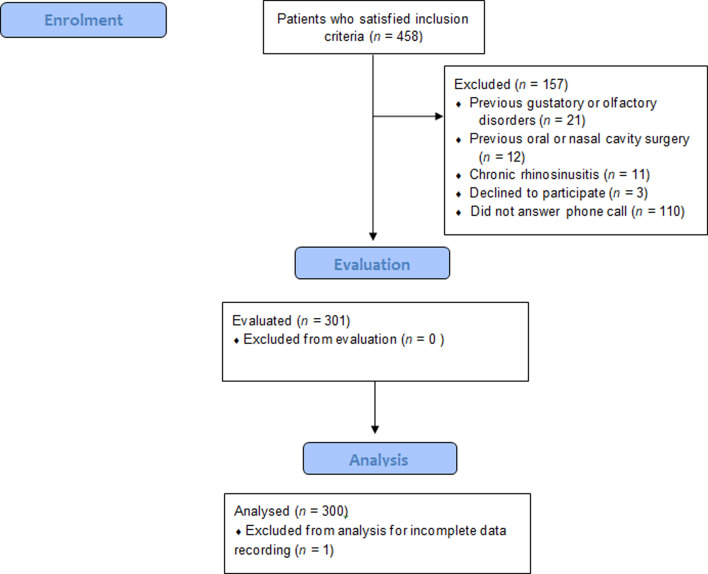
Consolidated Standards of Reporting Trials (‘CONSORT’) flowchart.

Of the 458 eligible patients, 157 were excluded in accordance with the exclusion criteria (Figure 1). The 301 remaining patients undertook the chemosensitive psychophysical test. One patient was excluded from the analysis because of an error in the recorded data. The general and clinical features of the patients are summarised in Table 3.

**Table 3.** General and clinical features of study population

Characteristic	Value
Gender (*n* (%))	
– Male	75 (25)
– Female	225 (75)
Age (mean ± SD (IQR); years)	43.6 ± 12.2 (33–53)
Reported symptoms (*n* (%))	
– Asymptomatic	28 (9.3)
– Fever	204 (68)
– Headache	133 (44.3)
– Myalgia	128 (42.7)
– Asthenia	109 (36.3)
– Cough	95 (31.7)
– Pneumonia	74 (24.7)
– Diarrhoea	70 (23.3)
– Nausea	35 (11.7)
– Dyspnoea	31 (10.3)
– Nasal obstruction	27 (9)
– Sore throat	24 (8)
– Conjunctivitis	11 (3.7)

SD = standard deviation; IQR = interquartile

The objective evaluation revealed that 210 patients (70 per cent) had a chemosensitive disorder. In 54.7 per cent of cases, both taste and smell were affected, 8.7 per cent of patients had isolated olfactory disorders, while in 6.7 per cent of cases only taste was affected (Table 4). Both the olfactory and gustatory disturbances detected were, in the majority of cases, severe. In fact, 47 per cent and 38 per cent of patients presented olfactory and gustatory scores indicative of complete anosmia and ageusia, respectively (Table 4).

**Table 4.** Chemosensory function assessment results

Parameter	Patients (*n* (%))
Chemosensory dysfunctions	
– Olfactory & taste disorders	164 (54.7)
– Only olfactory disorders	26 (8.7)
– Only taste disorders	20 (6.7)
– Total	210 (70)
– No taste or olfactory disorders	90 (30)
Olfactory dysfunctions	
– Anosmia	141 (47)
– Severe hyposmia	22 (7.3)
– Moderate hyposmia	19 (6.3)
– Mild hyposmia	8 (2.7)
– Normal	110 (36.7)
Gustatory dysfunctions	
– Ageusia	114 (38)
– Severe hypogeusia	22 (7.3)
– Moderate hypogeusia	30 (10)
– Mild hypogeusia	18 (6)
– Normal	116 (38.7)

The statistical significance of the associations between olfactory and gustatory disorders and some general and clinical characteristics of the patients was assessed. No significant correlations were found between the olfactory disturbances and the independent variables tested (Table 5). On the contrary, statistically significant correlations were found between taste dysfunctions and female gender (odds ratio = 1.936, 95 per cent CI = 1.139–3.386, *p* = 0.014) and fever (odds ratio = 2.132, 95 per cent CI = 1.299–3.499, *p* = 0.003) (Table 6).

**Table 5.** Olfactory logistic regression and cross-tabulation analysis results

Parameter	Olfactory dysfunction patients (*n* (%))	No olfactory dysfunction (*n* (%))	OR	95% CI for OR		Fisher's exact test value
				Lower limit	Upper limit	
Gender*:olfactory dysfunction^†^						
– Female	149 (66.2)	76 (33.8)	1.626	0.955	2.768	0.073
– Male	41 (54.7)	34 (45.3)				
Age*:olfactory dysfunction^†^						
– <50 years old	128 (65)	69 (35)	1.227	0.751	2.005	0.415
– ≥50 years old	62 (60.2)	41 (39.8)				
Fever*:olfactory dysfunction^†^						
– Fever	136 (71.6)	54 (28.4)	1.556	0.946	2.558	0.082
– No fever	68 (61.8)	42 (38.2)				
Headache*:olfactory dysfunction^†^						
– Headache	89 (66.9)	44 (33.1)	1.322	0.821	2.128	0.251
– No headache	101 (60.5)	66 (39.5)				
Pneumonia*:olfactory dysfunction^†^						
– Pneumonia	52 (70.3)	22 (29.7)	1.507	0.856	2.654	0.155
– No pneumonia	138 (61.1)	88 (38.9)				

*Dependent variable; ^†^independent variable. OR = odds ratio; CI = confidence interval

**Table 6.** Gustatory logistic regression and cross-tabulation analysis results

Parameter	Gustatory dysfunction patients (*n* (%))	No gustatory dysfunction (*n* (%))	OR	95% CI for OR		Fisher's exact test value
				Lower limit	Upper limit	
Gender*:gustatory dysfunction^†^						
– Female	147 (65.3)	78 (34.7)	1.936	1.139	3.286	0.014
– Male	37 (49.3)	38 (50.7)				
Age*:gustatory dysfunction^†^						
– <50 years old	123 (62.4)	74 (37.6)	1.144	0.703	1.863	0.587
– ≥50 years old	61 (59.2)	42 (40.8)				
Fever*:gustatory dysfunction^†^						
– Fever	137 (64.7)	67 (35.3)	2.132	1.299	3.499	0.003
– No fever	47 (49)	49 (51)				
Headache*:gustatory dysfunction^†^						
– Headache	82 (61.6)	51 (38.4)	1.102	0.919	1.636	0.919
– No headache	102 (61.1)	65 (38.9)				
Pneumonia*:gustatory dysfunction^†^						
– Pneumonia	48 (64.9)	26 (35.1)	1.222	0.707	2.11	0.473
– No pneumonia	136 (60.2)	90 (39.8)				

*Dependent variable; ^†^independent variable. OR = odds ratio; CI = confidence interval

## Discussion

Olfactory and gustatory disorders have been recognised as early symptoms of the SARS-CoV-2 infection.^1–12^ Their high frequency in Covid-19 patients makes them fundamental diagnostic markers, potentially enabling the early detection and isolation of suspect cases. Such health control measures will be crucial in the coming months in order to contain any new epidemic clusters.

This study adds to a very small number of other series that have evaluated chemosensitive functions in Covid-19 patients using psychophysical tests.^15–18^ The objective evaluation of these patients is essential in order to: quantify the exact frequency of chemosensitive disorders, obtain a standardised classification of severity and monitor their recovery over time. The major strength of the present study is the analysis of a large series of patients in an early stage of infection, when olfactory and gustatory disturbances are actually at their maximum severity and have not yet begun to regress. In fact, the studies already published, although objective, mostly evaluate patients two to three weeks after clinical onset. The lack of early objective data is linked to insurmountable logistical problems in evaluating these patients, who are often in home quarantine, and hence there is limited accessibility for the operators who should perform the test. These problems have been overcome by using a remote self-administered psychophysical test that was recently proposed and validated by our research team.^19^ Moreover, this test guarantees the operator's safety; this risk is another factor limiting the execution of psychophysical tests with infected patients.

The results of this study revealed a very high frequency of chemosensitive disorders in SARS-CoV-2 infected patients. As noted by other authors,^1,3,9,11,16–18^ in Covid-19, chemosensitive disorders are typically not associated with rhinitis symptoms and nasal obstruction, present only in 9 per cent of the patients included in this study. In 16.2 per cent of patients, the chemosensitive disorder was the first manifestation of the disease, while in 4.6 per cent it was the only manifestation.

The dysfunctions detected were principally complete anosmia and/or ageusia, or severe hyposmia and/or hypogeusia. Such a high rate of severe disorders can be ascribed to the very early stage of the Covid-19 in the patients evaluated, when functional recovery has not yet started.

The association between chemosensitive disorders and female gender has already been reported by other authors, and is hypothetically related to different immune response patterns between men and women.^9^ In this study, this relationship was detected only for taste, but the series presented an imbalance between males (25 per cent of the pool) and females (75 per cent). Further studies with more homogeneous cases will be necessary to confirm this result.

Interestingly, no significant correlation was found between olfactory disorders and headaches, and no other symptoms related to central nervous system involvement were reported by the patients. This finding, together with the tendency for spontaneous remission of the dysfunction within a few weeks,^9,14,16–19^ would suggest a pathogenesis linked to inflammatory damage in the olfactory epithelium. However, the ability of SARS-CoV-2 to invade the central nervous system causing neuronal damage cannot be excluded.^20^

Some authors have proposed an association between the presence of chemosensitive disorders and mild Covid-19.^21,22^ In our case history, there was no significant association between olfactory or gustatory disorders and the early relief of lung involvement. In considering these results, it should be clarified that not all the patients routinely underwent a computed tomography scan of the chest. Moreover, the time of pulmonary deterioration is generally longer than that observed in this study. Prospective objective studies will be necessary to clarify the prognostic value of chemosensitive disorders.^8^

• The proposed and validated psychophysical test is effective for fast, objective evaluation of early-stage coronavirus disease 2019 (Covid-19) patients
• Chemosensitive disorders are frequent, early symptoms of the coronavirus infection
• In a significant number of cases, chemosensitive disorders are the first or only manifestation of Covid-19
• This study establishes a solid objective base for prospective evaluation of functional recovery and the prognostic value of chemosensitive disorders

## Conclusion

The psychophysical test proposed and validated by Vaira *et al*.^19^ has proven to be an effective tool for the fast and objective evaluation of patients in the early stages of Covid-19. Based on our results, chemosensitive disorders have been confirmed to be frequent and early symptoms of the coronavirus infection, and in a significant number of cases they are the first or only manifestation of Covid-19. This study establishes a solid objective base for the prospective evaluation of the functional recovery and the prognostic value of chemosensitive disorders.
